# A taxonomic study of *Muscidifurax* Girault & Sanders from China (Hymenoptera, Chalcidoidea, Pteromalidae)

**DOI:** 10.3897/zookeys.776.25030

**Published:** 2018-07-26

**Authors:** Hui Xiao, Shi-yu Zhou, Yan-feng Tong

**Affiliations:** 1 Key Laboratory of Zoological Systematics and Evolution, Institute of Zoology, Chinese Academy of Sciences, Beijing 100101, China Institute of Zoology, Chinese Academy of Sciences Beijing China; 2 College of life sciences, Shenyang Normal University, Shenyang, Liaoning 110034, China Shenyang Normal University Liaoning China

**Keywords:** China mainland, key, *
Muscidifurax
*, new species, Pteromalidae, taxonomy

## Abstract

Five species of *Muscidifurax* Girault & Sanders (Hymenoptera: Pteromalidae) are studied from mainland China, of which three new species, *M.similadanacus* Xiao & Zhou, **sp. n.**, *M.sinesensilla* Xiao & Zhou, **sp. n.**, *M.neoraptorellus* Xiao & Zhou, **sp. n**., and one newly recorded species, *M.adanacus* Doganlar, are reported. All species have been reared from pupae of *Muscadomestica* Linnaeus. A key to Chinese *Muscidifurax* and illustrations of external features of the species are provided.

## Introduction

*Muscidifurax* was described by Girault and Sanders in 1910 to include *M.raptor* Girault and Sanders, parasitizing the common house fly (*Muscadomestica* Linnaeus) from Illinois, USA. The genus can be recognized by the female antenna with one anellus and seven funicular segments (two anelli and six funicular segments in male), head protuberant at level of antennal toruli, marginal vein thickened in proximal half and progressively thinner in distal half. Since then, several researchers have studied the genus, including Graham (1969), Dzhanokmen (1978) and Bouček (1991). Kogan and Legner (1970) studied the genus and described four new species from Nearctic region. Doganlar (2007) described a new species of *Muscidifurax* which probably parasites *Fannia* sp. Thus, six valid species are described in the genus. All species are parasitoids of species of Calliphoridae and Muscidae (Diptera). Some species, such as *M.raptor*, were used in the biological control of the house fly (Legner 1971; Doganlar 2007). Until now, only one species, *Muscidifuraxraptor* Girault and Sanders, has previously been recorded in China.

## Materials and methods

All specimens were collected in the laboratory where they have been reared from pupae of house flies, and preserved in 75% ethanol. They were subsequently air-dried, point-mounted, and examined with a LEICA M10 stereomicroscope. Photographs were taken by using a Nikon Multizoom AZ100 system, and plates of illustrations were compiled using Adobe Photoshop® software. Five species have been identified, including three new species (*M.similadanacus* sp. n., *M.sinesensilla* sp. n., *M.neoraptorellus* sp. n.) and one newly recorded species (*M.adanacus* Doganlar). All type specimens are deposited in the Institute of Zoology, Chinese Academy of Sciences, Beijing, China (IZCAS).

Morphological terminology follows that of Graham (1969), Bouček (1988), and Gibson et al. (1997). All specimens were examined and identified based on the studies of Kogan and Legner (1970), Doganlar (2007) and Girault and Sanders (1910). Body length (i.e. the length of body excluding the ovipositor sheaths) is measured in millimeters (mm), other measurements are given as ratios.

Abbreviations of morphological terms used are:

**Fu_n_** funicular segment number;

**POL** posterior ocellar distance;

**OOL** ocellocular distance;

**Gt_n_** gastral tergite number.

## Taxonomy

### Key to species

**Table:** 

1	Fore wing without marginal fringe and usually with reduced pilosity (Fig. 6); inner margins of eyes not angularly produced upwards near vertex	**2**
–	Fore wing with marginal fringe well developed, or at least with marginal fringe at posterior margin (Fig. 12); inner margins of eyes angularly produced upwards (small angle shape) near vertex (Figs 9, 14)	**4**
2	Second funicular segment without sensilla (Fig. 24); gaster 1.65× as long as broad, Gt_1_ about 1/3 length of gaster; median area of propodeum without coarse rugae	***M.neoraptorellus* sp. n.**
–	Second funicular segment with sensilla; gaster at least 1.9× as long as broad; Gt_1_ about 1/4 length of gaster; median area of propodeum with weak or strong coarse rugae	**3**
3	Each funicular segment longer than broad; head width 1.25× head height; Fu_1_ slightly longer than Fu_2_; propodeum with distinct costula (Fig. 5)	***M.similadanacus* sp. n.**
–	Fu_1_-Fu_5_ or Fu_1_-Fu_6_ longer than broad, Fu_7_ quadrate; head width 1.17× head height; Fu_1_ shorter or as long as Fu_2_; propodeum without costula (Fig. 19)	***M.adanacus* Doganlar**
4	Antennal insertion under the lower ocular line, Fu_1_ without sensilla (Fig. 10); head 1.82× as broad as long in dorsal view; propodeum without coarse rugae; gaster 1.8× as long as broad	***M.sinesensilla* sp. n.**
–	Antennal insertion on the lower ocular line, Fu_1_ with sensilla; head 2× as broad as long in dorsal view; propodeum with coarse rugae; gaster 2× as long broad	***M.raptor* Girault & Sanders**

### Muscidifurax

Taxon classificationAnimaliaHymenopteraPteromalidae

Girault & Sanders, 1910

Muscidifurax Girault & Sanders, 1910: 146.
Muscidifurax raptor Girault & Sanders, 1910: 146; original designation and monotypy. Kogan and Legner 1970: 1268–1290; Propp 1984: 705; Narendran et al. 2006: 29–34. [Type species.]
Smeagolia Hedqvist, 1973: 237. Type species: Smeagoliaperplexa Hedqvist. Synonymized by Bouček 1991: 203.

#### Diagnosis.

Body dark green, head, and mesosoma with distinctly white hairs, eye glabrous. Head wider than mesosoma, occipital carina strong. Antennal insertion placed on lower ocular line and face distinctly protuberant at antennal insertion; lower face receding almost horizontally. Antenna slender, formula 11173 in females, 11263 in males; lower margin of clypeus more or less incised medially, without median tooth. Pronotal collar margined; notauli incomplete; scutellum flattened; propodeum with median carina and complete plicae, nucha short but distinct. Marginal vein strongly thickened in proximal half (its lower margin distinctly sinuate) and progressively thinner in distal half. Gaster flattened dorsally, hind margin of Gt_1_ trilobed.

#### Biology.

Hosts include Calliphoridae (*Chrysomya* sp., *Phormia* sp.) and Muscidae (*Fanniacanicularis*, *Fanniafemoralis*, *Muscadomestica*, *Stomoxys* sp. and *Stomoxyscalcitrans*) (Noyes 2017).

#### Distribution.

Palaearctic, Nearctic, Afrotropics, Neotropics, and Australasian regions (Noyes, 2017). China: Beijing, Shandong (Guo et al. 1997).

### Muscidifurax similadanacus

Taxon classificationAnimaliaHymenopteraPteromalidae

Xiao & Zhou sp. n.

http://zoobank.org/24C8BB2B-9695-455A-AEB8-7F57EC21BB90

Figs 1–7

#### Diagnosis.

Fore wing without marginal fringe; each funicular segment longer than broad; head width 1.25× head height; Fu_1_ slightly longer than Fu_2_; Fu_1_ without sensilla; median area of propodeum with distinct costula; gaster 2.1× as long as broad, Gt_1_ 1/4 length of gaster.

#### Description.

**Figures 1–7.**
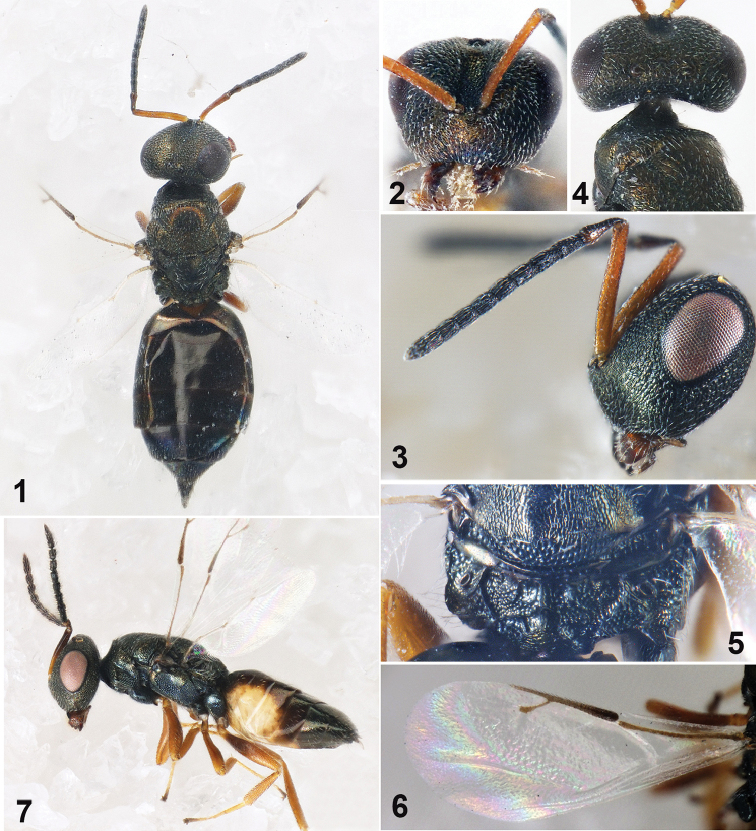
*Muscidifuraxsimiladanacus* sp. n., **1–6** female holotype **1** Body in dorsal view **2** Head in frontal view **3** Head in lateral view **4** Head in dorsal view **5** Propodeum 6 Fore wing **7** Male, Body in lateral view.

Holotype. *Female*. 3.4 mm (Fig. 1). Head and mesosoma dark green, with metallic reflections and white hairs; gaster brown with yellow spot. Antennal scape brown, flagellum dark brown; legs yellow except coxae concolorous with body, femora and pretarsi brown; fore wings hyaline, venation brown except marginal vein dark brown.

Head in frontal view 1.25× as wide as high (Fig. 2); eyes with inner margins parallel, eye height 0.6× head height, eyes separated by 1.5× their height; antennal scrobes deep, reaching anterior ocellus. Antennal insertion on lower ocular line. Clypeal margin slightly protruded, straight; oral fossa 0.44× as wide as head; right mandible with four teeth, left mandible with three teeth. Head in lateral view with malar sulcus inconspicuous, eye height 1.74× malar space. Antennal scape length 1.44× eye height, exceeding vertex (Figs 2, 3); pedicel in lateral view 2.38× as long as broad; anellus transverse; Fu_1_ 1.8× as long as broad, slightly longer than Fu_2_; each funicular segment with sensilla except Fu_1_; clava not clavate, 2.25× as long as broad. Head in dorsal view (Fig. 4), 1.82× as wide as long; vertex convex; eye length 2.86× temple length; POL 1.33× OOL.

Head as broad as mesosoma. Mesosoma not distinctly convex, 2.13× as long as broad. Pronotum 0.85× as broad as mesoscutum, anteriorly margined, posterior band smooth. Mesoscutum 1.74× as broad as long, anterior half weakly reticulate and posterior half with deep reticulation; notauli incomplete, only distinct basally. Scutellum 1.18× as broad as long, frenal line absent; reticulation shallow. Propodeum (Fig. 5) medially 0.6× as long as scutellum, reticulation irregular on median area, with short irregular carinae; plicae distinct and complete, separated by 1.2× medial length of propodeum; median carina complete, costula distinct; nucha short; propodeal spiracles oval, 1.5× as long as broad. Fore wing 2.53× as long as broad, without marginal fringe (Fig. 6); basal vein and basal cell bare; upper surface of costal cell bare, lower surface with scattered setae; submarginal vein 1.75× marginal vein, marginal vein 1.8× postmarginal vein, postmarginal vein longer than stigmal vein (1.33×); stigmal vein slightly capitate.

Gaster spindle-shaped (Fig. 1) with apex pointed, 2.1× as long as broad, 1.49× as wide as mesosoma; Gt_1_ covering 1/4 of gaster, each segment with hind margin entire except hind margin of Gt_1_ trilobed.

*Male*. As female, with the following differences. Body length 3.0–3.5 mm (Fig. 7). Antennal insertion above the lower ocular line, each funicular segment longer than broad, with 3–4 rows setae.

#### Variability.

Females: body length 2.9–3.5 mm, others same as holotype. Males: body length 2.6–3.0 mm.

#### Remarks.

This new species is similar to *M.raptor* and *M.sinesensilla* sp. n., but noticeably different by the absence of a marginal fringe on the fore wing. It is also very close with *M.adanacus* in having the fore wing without a fringe, but can be recognized with the characters listed in the key.

#### Material examined.

Holotype. ♀, China: Xinjiang: Urumqi, 43.45°N 87.36°E, VII.2016, ex. Pupa of *Muscadomestica*, leg. Hao-yuan Hu, IOZ(E)1812530 (2016-WJ-066). Paratypes. 7♂, IOZ(E)1812531-1812537 (2016-WJ-062), 7♀, IOZ(E)1812538-1812544(2016-WJ-066), same data to holotype.

#### Etymology.

The name refers to the similarity of this species with *M.adanacus*, and is to be treated as an adjective.

#### Hosts.

Pupa of *Muscadomestica*.

#### Distribution.

China (Xinjiang).

### Muscidifurax sinesensilla

Taxon classificationAnimaliaHymenopteraPteromalidae

Xiao & Zhou sp. n.

http://zoobank.org/AAD25D3A-2FC7-4695-ACB0-208CBED8C1FD

Figs 8–14

#### Diagnosis.

Fore wing with marginal fringe; inner margins of eyes angularly produced upwards (small angle shape) near the vertex; Fu_1_ without sensilla; head 1.82× as broad as long dorsally; propodeum without coarse rugae; gaster 1.8× as long as broad.

#### Description.

**Figures 8–14.**
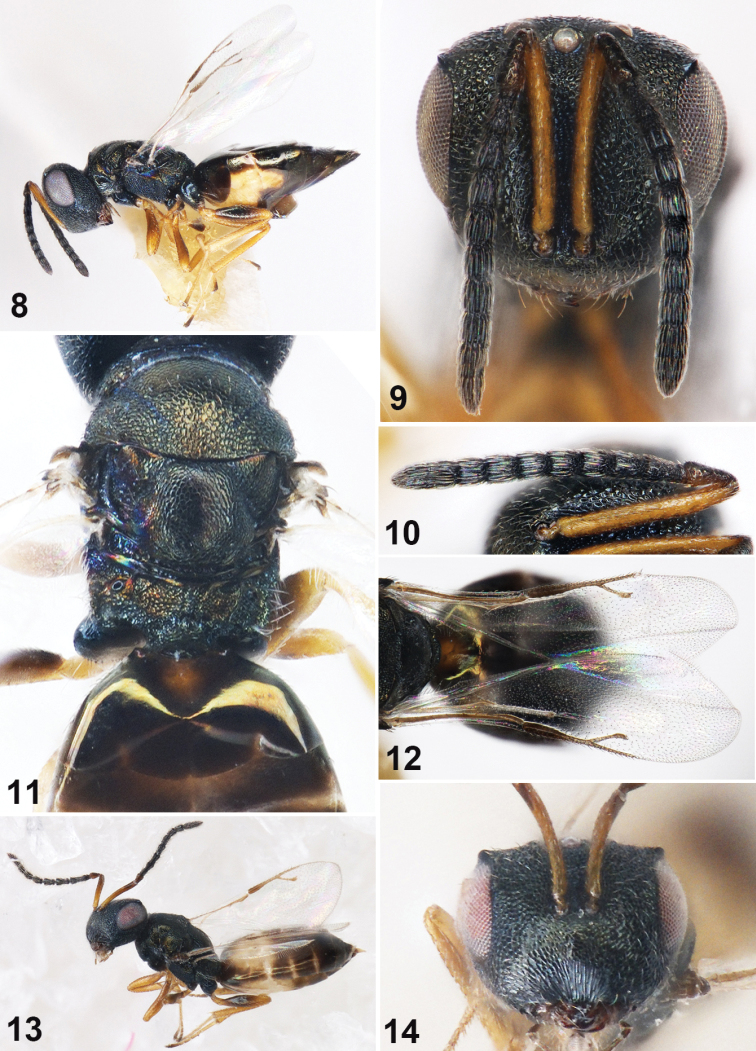
*Muscidifuraxsinesensilla* sp. n., **8–12** female holotype **8** Body in lateral view **9** Head in frontal view **10** Head in lateral view **11** Propodeum **12** Fore wing **13–14** Male **13** Body in lateral view **14** Head in frontal view.

Holotype. *Female*. 2.5 mm (Fig. 8). Head and mesosoma dark blue, with metallic reflections; gaster brown with yellow spot. Antennal scape yellowish brown, flagellum dark brown; legs yellow except coxae concolorous with body, and femora and pretarsi brown; fore wings hyaline, venation brown except marginal vein dark brown.

Head in frontal view 1.17× as wide as high; inner margins of eyes angularly produced upwards (small angle shape) near the vertex (Fig. 9); eye height 0.54× head height, eyes separated by 1.53× their height; antennal scrobes deep, not reaching anterior ocellus; reticulation in antennal scrobe smaller than that on parascrobe. Antennal insertion on lower ocular line, distance from upper margin of torulus to lower margin of anterior ocellus 1.78× distance from lower margin of torulus to lower margin of clypeus. Clypeus with longitudinal striation; clypeal margin slightly protruded, straight; oral fossa 0.5× as wide as head; right mandible with four teeth, left mandible with three teeth. Head in lateral view with malar sulcus conspicuous, eye height 1.25× malar space. Antennal scape length 1.34× eye height, reaching anterior ocellus, but not exceeding vertex; length of flagellum and pedicel combined longer than head width (1.2×); pedicel in lateral view 2× as long as broad; anellus transverse; Fu_1_ 1.67× as long as broad, slightly longer than Fu_2_; each funicular segment with sensilla except Fu_1_ (Fig. 10); clava not clavate, 2.35× as long as broad. Head in dorsal view, 1.82× as wide as long; vertex convex; eye length 2.55× temple length; POL 0.76× OOL.

Head 1.04× as broad as mesosoma. Mesosoma not distinctly convex, 1.41× as long as broad. Pronotum 0.74× as broad as mesoscutum, anteriorly margined, posterior band smooth and with a row of hairs. Mesoscutum 1.91× as broad as long; notauli incomplete, only distinct basally. Scutellum with reticulation shallow, frenal line absent. Propodeum (Fig. 11) medially 0.65× as long as scutellum, reticulation irregular; plicae complete, separated by 1.23× medial length of propodeum; median carina raised and complete; nucha short; propodeal spiracles oval, 1.5× as long as broad. Fore wing 2.35× as long as broad, with marginal fringe (Fig. 12); basal vein and basal cell bare; upper surface of costal cell hairy, lower surface with scattered setae; submarginal vein 1.32× marginal vein, marginal vein 1.82× postmarginal vein, postmarginal vein longer than stigmal vein (1.3×); stigmal vein straight, stigmal slightly capitate.

Gaster sessile, spindle-shaped with apex pointed, 1.8× as long as broad, 1.45× as wide as thorax; each segment with hind margin entire except hind margin of Gt_1_ trilobed.

*Male*. As female, with the following differences. Body length 2.0 mm (Fig. 13). Antennal insertion above the lower ocular line (Fig. 14), Fu_1_ 0.44× as long as scape, each funicular segment longer than broad, with 3–4 rows of setae.

#### Variability.

Females: body length 2.3–2.5 mm, others same as holotype. Males: body length 1.4–2.2 mm.

#### Remarks.

This new species is very similar to *M.raptor* having fore wing with marginal fringe and inner margins of eyes angularly produced upwards near the vertex. It differs from *M.raptor* in having the first funicular segment without sensilla, propodeum without coarse rugae.

#### Material examined.

Holotype. ♀, China: Xinjiang: Urumqi, 43.45°N 87.36°E, VII.2016, ex. Pupa of *Muscadomestica*, leg. Hao-yuan Hu, IOZ(E)1812546 (2016-WJ-044). Paratypes. 7♂, IOZ(E)1812547-1812553(2016-WJ-045), 2♀, IOZ(E)1812554-1812555(2016-WJ-044), same data to holotype.

#### Etymology.

The specific name is derived from the Latin *sine*- and *sensilla*, referencing the character of Fu_1_ without sensilla. The name is to be treated as a noun in apposition.

#### Hosts.

Pupa of *Muscadomestica*.

#### Distribution.

China (Xinjiang).

### Muscidifurax adanacus

Taxon classificationAnimaliaHymenopteraPteromalidae

Doganlar, 2007

Figs 15–21

Muscidifurax adanacus Doganlar, 2007: 245–246. Holotype ♀, MKUT. Not examined.

#### Diagnosis.

**Figures 15–21.**
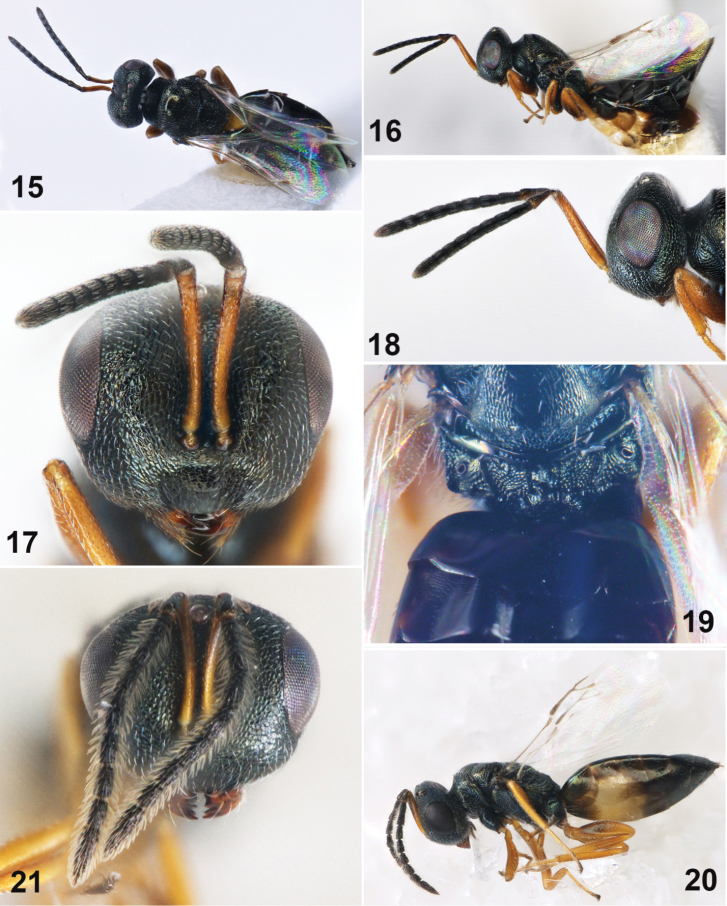
*Muscidifuraxadanacus* Doganlar, 2007, **15–19** female **15** Body in dorsal view **16** Body in lateral view **17** Head in frontal view **18** Head in lateral view **19** Propodeum **20–21** Male **20** Body in lateral view **21** Head in frontal view.

Antenna with scape longer than eye height (Figs 16, 17), exceeding vertex; each funicular segment longer than broad except Fu_7_ subquadrate; Fu_1_ without sensilla, longer than Fu_2_ (Fig. 18); Fu_2_ with sensilla. Propodeum with two slim median carinae, plicae present, nucha developed (Fig. 19); median area of propodeum with weakly or strong coarse rugae. Fore wing without marginal fringe, and with reduced pilosity. Gaster at least 1.9× as long as broad; Gt_1_ about 1/4 length of gaster (Fig. 15). Male antennae with each funicular segment longer than broad, and with dense hairy (Figs 20, 21).

#### Material examined.

China: 1♂ (2016-WJ-067), 4♀ (2016-WJ-004), Shandong: Jinan, 22.III.2016, reared from pupa of *Muscadomestica* (captured on 27.II.2016), leg. Zhang-ze Hu.

#### Hosts.

Pupa of *Muscadomestica*.

#### Distribution.

China (Shandong); Palearctic region (Turkey).

### Muscidifurax neoraptorellus

Taxon classificationAnimaliaHymenopteraPteromalidae

Xiao & Zhou sp. n.

http://zoobank.org/81DADF11-ADE6-45B4-A668-FFEBB95392A7

Figs 22–27

#### Diagnosis.

Clypeus with longitudinal striation; clypeal margin not protruded; antenna with each funicular segment longer than broad, each funicular segment with sensilla except Fu_1_ and Fu_2_; median area of propodeum without coarse rugae; fore wing without marginal fringe, usually with reduced pilosity; gaster 1.65× as long as broad, Gt_1_ 1/3 length of gaster.

#### Description.

**Figures 22–27.**
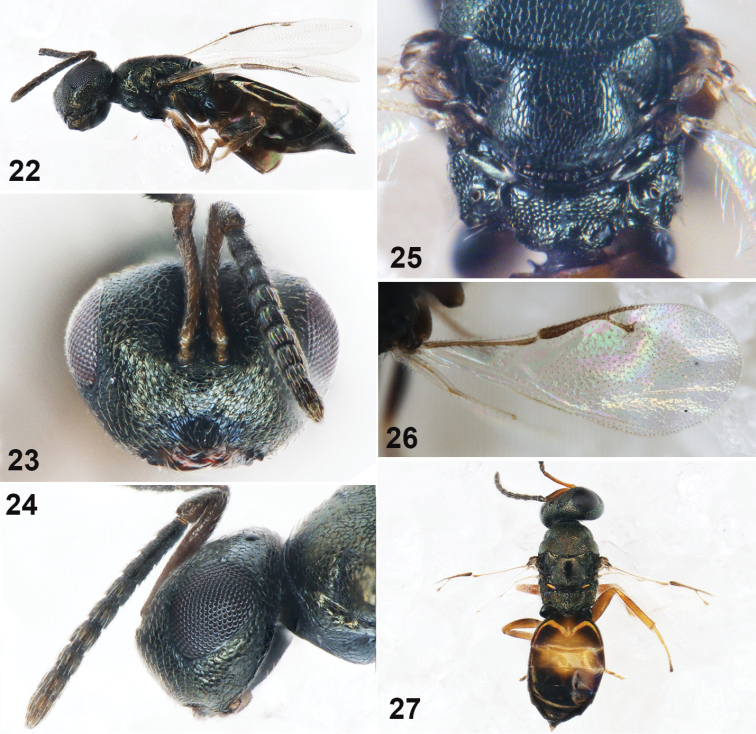
*Muscidifuraxneoraptorellus* sp. n., **22–26** female holotype **22** Body in lateral view **23** Head in frontal view **24** Head and antenna in lateral view **25** Propodeum **26** Fore wing **27** Male, Body in dorsal view.

Holotype. *Female*. 2.2 mm (Fig. 22). Head and mesosoma black, with blue metallic reflections; gaster dark brown with metallic reflections basally. Antennal scape brown, flagellum dark brown; legs brown except coxae concolorous with body; fore wings hyaline, venation brown except marginal vein dark brown.

Head in frontal view 1.13× as wide as high (Fig. 23); eye height 0.54× head height, eyes separated by 1.63× their height; antennal scrobes deep, not reaching anterior ocellus; reticulation in antennal scrobe smaller than that on parascrobe. Antennal insertion on lower ocular line, distance from upper margin of torulus to lower margin of anterior ocellus 1.56× distance from lower margin of torulus to lower margin of clypeus. Clypeus with longitudinal striation; clypeal margin straight, not protruded; oral fossa 0.46× as wide as head; right mandible with four teeth, left mandible with three teeth. Head in lateral view (Fig. 24) with malar sulcus inconspicuous, eye height 1.24× malar space. Antennal scape length 1.33× as long as broad, reaching vertex; length of flagellum and pedicel combined longer than head width (1.28×); anellus transverse; each funicular segment longer than broad, Fu_1_ 1.38× as long as broad, as long as Fu_2_; each funicular segment with sensilla except Fu_1_ and Fu_2_ (Fig. 24); clava not clavate, 2.67× as long as broad. Head in dorsal view, 1.75× as wide as long; vertex convex and with coarse reticulation; eye length 2.47× temple length; POL 0.75× OOL.

Head as broad as mesosoma. Mesosoma 1.33× as long as broad. Pronotum 0.83× as broad as mesoscutum, anteriorly margined, posterior band smooth and with a row of hairs. Mesoscutum 1.83× as broad as long; notauli only distinct basally. Scutellum with reticulation shallow, frenal line absent. Propodeum (Fig. 25) medially 0.8× as long as scutellum, reticulation irregular; plicae distinct and complete, separated by 1.44× medial length of propodeum; median carina raised and complete; nucha short, with coarse reticulation; propodeal spiracles oval. Fore wing 2.62× as long as broad, without marginal fringe (Fig. 26); basal vein and basal cell bare; submarginal vein 1.37× marginal vein, marginal vein 1.73× postmarginal vein, postmarginal vein longer than stigmal vein (1.32×); stigmal vein straight, stigmal slightly capitate.

Gaster sessile, spindle-shaped with apex pointed, 1.65× as long as broad, 1.14× as wide as mesosoma; each segment with hind margin entire except Gt_1_ trilobed; Gt_1_ covering 1/3 length of gaster.

*Male*. As female, with the following differences. Body length 2.5 mm. Antennal insertion above the lower ocular line, each funicular segment longer than broad; Fu_1_ 0.5× as long as scape, longer than other funicular segments, 2.46× as long as wide. Lateral panel of metanotum golden (Fig. 27). Gaster dorsum yellow in median area.

#### Remarks.

This new species is very close to *M.raptorellus*, but noticeably different from *M.raptorellus* in having the first and second funicular segments without sensilla (only Fu_1_ without sensilla in *M.raptorellus*), and the median area of propodeum without coarse rugae (with distinctly coarse rugae in *M.raptorellus*).

#### Material examined.

Holotype. ♀, China: Shandong: Jinan, 36.40°N 117.00°E, 22.III.2016, reared from pupa of *Muscadomestica* (captured on 27.II.2016), leg. Zhang-ze Hu, IOZ(E)1812557 (2016-WJ-002). Paratypes. 1♂, IOZ(E)1812559 (2016-WJ-005), 1♀, IOZ(E)1812558 (2016-WJ-002), same data as holotype.

#### Etymology.

The species is intended to show similarities with *M.raptorellus*, hence the specific name is compound of ‘*neo*-’ and ‘*raptorellus*’. It is to be treated as an adjective.

#### Hosts.

Pupa of *Muscadomestica*.

#### Distribution.

China (Shandong).

### Muscidifurax raptor

Taxon classificationAnimaliaHymenopteraPteromalidae

Girault & Sanders, 1910

Muscidifurax raptor Girault & Sanders, 1910: 146; Doganlar 2007: 243–252.
Smeagolia perplexa Hedqvist, 1973: 237; Bouček, 1991: 203 (synonymy).

#### Diagnosis.

Body black green. Head 2× as long as broad in dorsal view. Antennal scrobes deep, extending upwards and not reaching anterior ocellus; clypeus with shallowly longitudinal striation, lower margin slightly protruded. Antenna with each funicular segment longer than broad and with sensilla. Propodeum with plicae distinct and complete, median carina raised and complete; costula distinct. Fore wing with marginal fringe; stigmal vein straight, slightly capitate. Gaster 2× as long as broad, slightly broader than mesosoma width; Gt_1_ covering 1/3 length of gaster.

#### Material examined.

China: 1♂, 2♀, Shandong: Jinan, 22.III.2016, reared from pupa of *Muscadomestica* (captured on 27.II.2016), leg. Zhang-ze Hu (2016-WJ-003); 1♀, Australia, N.S.W. Sydney, 10.I.1984, leg. R. Rilansow, det. B.R. Subba Rao, 1985.

#### Hosts.

Pupa of *Muscadomestica*.

#### Distribution.

China (Beijing, Shandong) (Guo et al. 1997); Afrotropics, Australasian, Nearctic, Neotropics and Palearctic regions.

## Supplementary Material

XML Treatment for Muscidifurax

XML Treatment for Muscidifurax similadanacus

XML Treatment for Muscidifurax sinesensilla

XML Treatment for Muscidifurax adanacus

XML Treatment for Muscidifurax neoraptorellus

XML Treatment for Muscidifurax raptor
